# Supplementary material for: Schizophrenia-Like Behavioral Impairments in Mice with Suppressed Expression of Piccolo in the Medial Prefrontal Cortex
Source: J Pers Med. 2021 Jun 26;11(7):607. doi: 10.3390/jpm11070607 (PMC8304324; doi:10.3390/jpm11070607)
Supplement: Supplementary file 1 [file jpm-11-00607-s001.zip › jpm-1240514-supplementary.pdf]

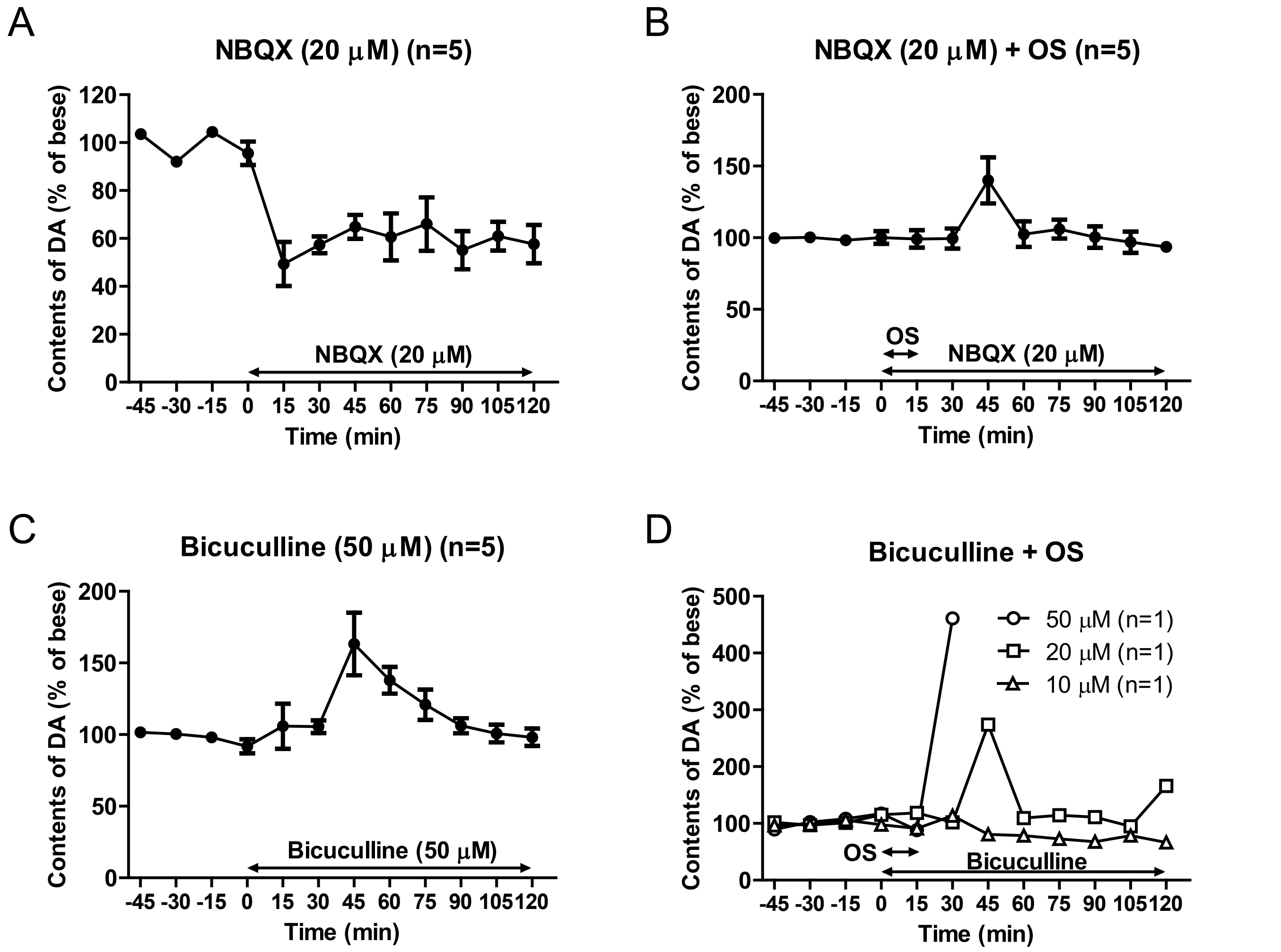

Supplementary Figure S1. *In vivo* antagonism of glutamate and GABA receptor on extracellular DA in microdialysis. (A) The average of DA levels in wildtype mice (n=5) in response to the application of NBQX (20 μM) is represented in the graph. (B) The average of DA levels in wildtype mice (n=5) in response to the application of NBQX (20 μM) and optical stimulation is represented in the graph. (C) The average of DA levels in wildtype mice (n=5) in response to the application of bicuculline (50 μM) is represented in the graph. (D) DA levels in each wildtype mouse in response to the application of bicuculline (10, 20 and 50 μM) and optical stimulation is represented in the graph. The mouse received bicuculine (50 μM) died just after the collection of 30-min fraction.

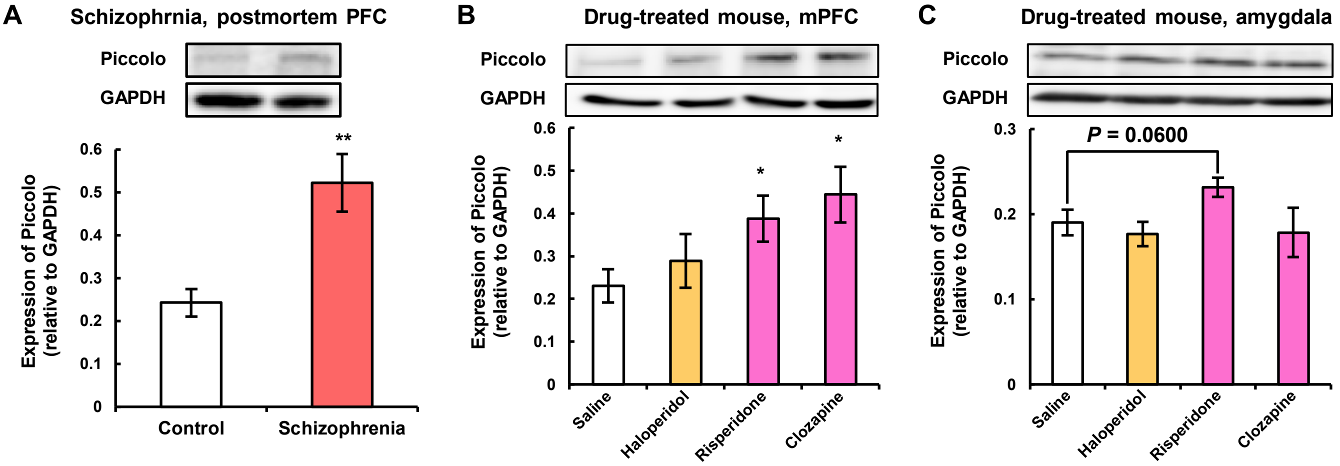

Supplementary Figure S2. Piccolo expression in the PFC of schizophrenia patients and of antipsychotic drug-treated mice

(*A*) Expression levels of Piccolo in the PFC of schizophrenia patients were measured by Western blotting and presented as relative to the expression levels of GAPDH (Control = 5, Schizophrenia = 8). $^{**}p < 0.01$ vs. Control (Student's *t*-test). (*B, C*) Expression levels of Piccolo in the mPFC (*B*) or the amygdala (*C*) of mice treated with antipsychotic drug for 14 days were measured by Western blotting and presented as relative to the expression levels of GAPDH (n = 5). $^{*}p < 0.05$ vs. Saline (Student's *t*-test).
